# Supplementary material for: Modulation of Fatty Acid Composition of Aspergillus oryzae in Response to Ethanol Stress
Source: Microorganisms. 2019 Jun 1;7(6):158. doi: 10.3390/microorganisms7060158 (PMC6616634; doi:10.3390/microorganisms7060158)
Supplement: Supplementary file 1 [file microorganisms-07-00158-s001.pdf]

Table S1. Primers used in quantitative real-time PCR.

Primers	5′-3′
GAPDH-F	ACCGCGGTTCATGCTTATAC
GAPDH-R	GATCGACATTCCGGTCACTT
18S rRNA-F	TTACCCAATCCCGACAC
18S rRNA-R	CCAGACTTGCCCTCCA
d9d1-F	CCCTGGTCCTGTCAGTCGTA
d9d1-R	CACGCTGTATGGGTCAAGGT
d9d2-F	CGTCATAACCGCCTTCGTAA
d9d2-R	TGTTGGATGCGACCTTTTTC
d12d-F	CCCTGCTAGTGCCGTACTTC
d12d-R	GATGGGAGTCTCCTCCATCA

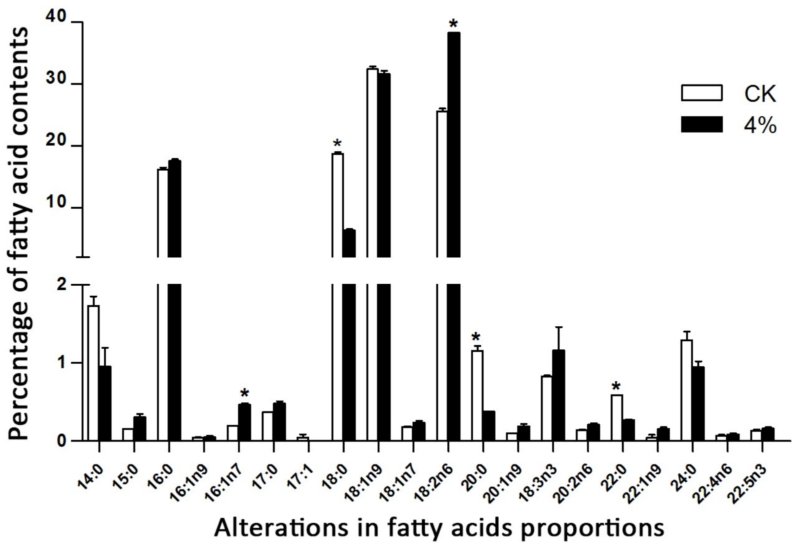

Figure S1. Alterations in the intracellular fatty acid proportions in response to 4% ethanol 36 h post-treatment. The bars represent the average (±SE) of the biological repeats. The asterisks indicate statistically significant differences between groups (Student's *t*-test): * denotes $P < 0.05$.
